# Treatment of Femoroacetabular Impingement with Surgical Dislocation

**DOI:** 10.4055/cios.2009.1.3.146

**Published:** 2009-08-17

**Authors:** Ho-Hyun Yun, Won-Yong Shon, Ji-Yeol Yun

**Affiliations:** Department of Orthopedic Surgery, Guro Hospital, Korea University School of Medicine, Seoul, Korea.

**Keywords:** Hip, Femoroacetabular impingement, Surgical dislocation

## Abstract

**Background:**

The authors report the results of femoroacetabular impingement (FAI) treated with a surgical dislocation.

**Methods:**

From April 2005 to May 2007, 15 FAI hips were treated with a surgical dislocation. The male/female ratio, mean age and mean symptom duration was 12/2, 35.8 years and 2.3 years, respectively. Radiographs and MR arthrograms were taken. The clinical evaluation involved changes in the pre- and postoperative Harris hip score (HHS).

**Results:**

There were 12 hips (80%) with at least one structural abnormality in the radiographs, with 11 (79%) labral tears and 8 (73%) abnormally high angles in the MR arthrograms. We performed 15 osteochondroplasties, 12 labral repairs, 12 acetabuloplasty, and 3 debridements. The mean HHS improved from 76 to 93 points. Three non-unions of the trochanteric osteotomy sites were encountered as complications.

**Conclusions:**

Radiographs and MR arthrograms are important for making a proper diagnosis of FAI and planning treatment. A surgical dislocation can be used to treat FAI but further technical improvements will be needed for fixation of the greater trochanteric osteotomy sites.

Femoroacetabular impingement (FAI) is a condition that has been recently recognized to generate hip pain.1) It occurs in patients with structural abnormalities of the femoral neck and acetabulum^1^,2) when they are involved in daily activities, sports, and occupations that require repetitive and constant flexion, adduction, and internal rotation of the hip joint. Repetitive micro damage and traction resulting from chronic FAI cause changes in the labrum (tear, degeneration, cyst formation, and ossification), as well as damage to the articular cartilage and subchondral bone, which eventually leads to early degenerative arthritis.^3^-7)

A patient interview, physical examination, radiographs, CT scan, and MRI (MR arthrogram) are normally used to diagnose FAI.8) In the interview and physical examination, pain and the limited range of movement are observed by internal rotation and flexion of the hip. An impingement test is also an effective diagnostic tool for these symptoms.9) FAI is also characterized by the radiographic findings of degenerative arthritis, acetabular retroversion, abnormal femoral head-neck junction (pistol-grip deformity), impingement findings between the anterosuperior acetabulum and the anterolateral femoral neck (abnormal ossification, bump, and herniation pit). Various measurement methods10) can be helpful for diagnosis but their accuracy, reproducibility, and efficacy are controversial. Acetabular retroversion and bony protuberance at the anterolateral femoral head-neck junction can be observed in a CT scan. However, MRI, particularly a MR arthrogram, is needed to identify an intra-articular lesion (a labral lesion and an articular cartilage injury.^11^-15)

The treatment methods for FAI include osteotomy,16) arthroscopy,^17^-19) arthroscopy and limited open techniques,^20^,21) as well as surgical dislocation.^7^,^19^,^22^,23) Osteotomy can be effective in widening the distance between the femoral neck and acetabulum in patients with Legg-Calvé-Perthes disease, avascular necrosis of the femoral head, or a femoral neck fracture accompanied by FAI as a complication.16) Arthroscopy is minimally invasive but difficult to perform, has a long learning-curve, and allows a resection of bony protrusions at the femoral head-neck junction. However, additional surgery is required when acetabuloplasty is insufficient due to difficulties in resecting the acetabulum and repairing a labral tear.24) Arthroscopy and limited open techniques are believed to combine the advantages of arthroscopy with those of a surgical dislocation but they may lengthen the surgery time and damage the lateral femoral cutaneous nerve. In addition, there are few reports on the clinical outcomes of the procedure. A surgical dislocation is relatively easy to perform with a large field of view but may result in complications caused by large procedures, including an osteotomy of the greater trochanter.25) Therefore, there are no established rules or guidelines for the choice of surgical technique.

In the present study the authors examined the outcomes of a surgical dislocation performed on patients with FAI.

## METHODS

Sixteen patients (17 cases) with FAI were treated with a surgical dislocation between April 2005 and May 2007 at our hospital. Among them, 14 patients (15 cases), who were available for a clinical and radiological follow-up of more than one year, were enrolled in this study. There were 12 males (86%) and 2 females (14%) with a mean age of 35.8 years (range, 22 to 54 years). The mean follow-up period was 2.3 years (range, 1 to 10 years).

A patient interview, physical examination, radiographs, and MR arthrogram were used to diagnose FAI. In history taking, there were no underlying diseases of the hip joint, experience of surgery, or obvious history of trauma. In the interview, the patients complained of discomfort, pain, and limited mobility during at least one of the following daily activities: sports activities, long-distance ambulation, entering and alighting vehicles, and standing up after 30 minutes of sitting.26) The physical examination revealed active hip flexion restricted to < 90° due to pain as well as pain and limited mobility during passive internal rotation and flexion of the hip. FAI was suspected when the anterior impingement test was positive. A MR arthrogram was performed to make a definite diagnosis.

**Fig. 1:**
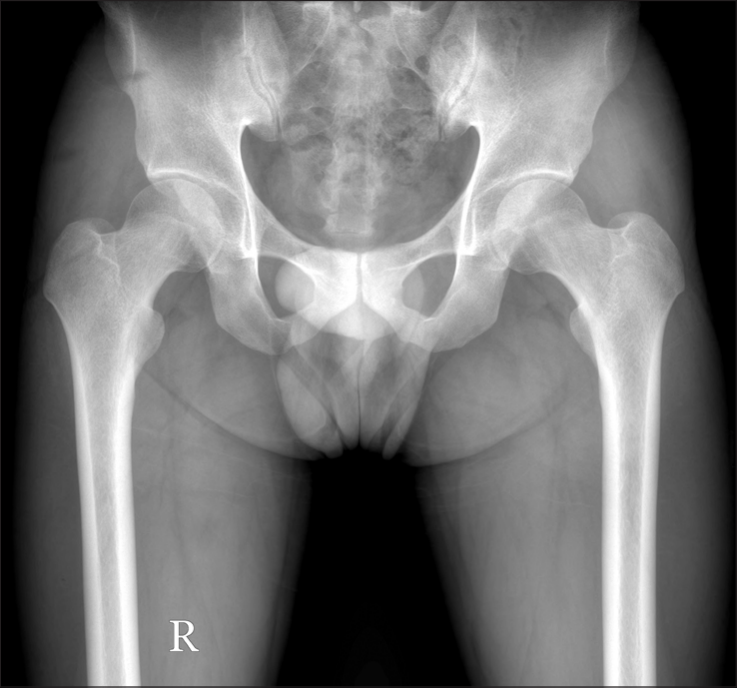
Both hip anteroposterior radiograph of a 25-year old male who suffered from left hip joint pain for 3 years shows a positive crossover sign in both hip areas.

**Fig. 2:**
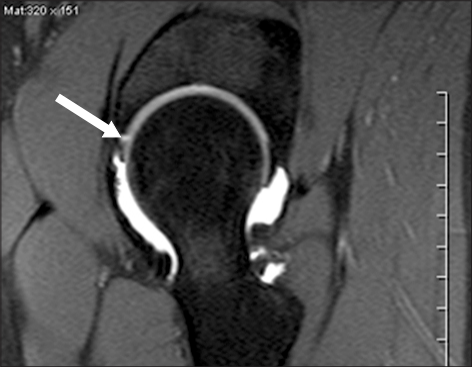
MR arthrogram in the T1-weighted image shows a labral tear at the anterosuperior portion.

The indications for a surgical dislocation were as follows: a ≥ 1 year duration of symptoms, no response to 2-3 months of conservative treatments (Fig. 1), and a labral lesion (tear and a severe degenerative change) on the MR arthrogram (Fig. 2).

**Fig. 3:**
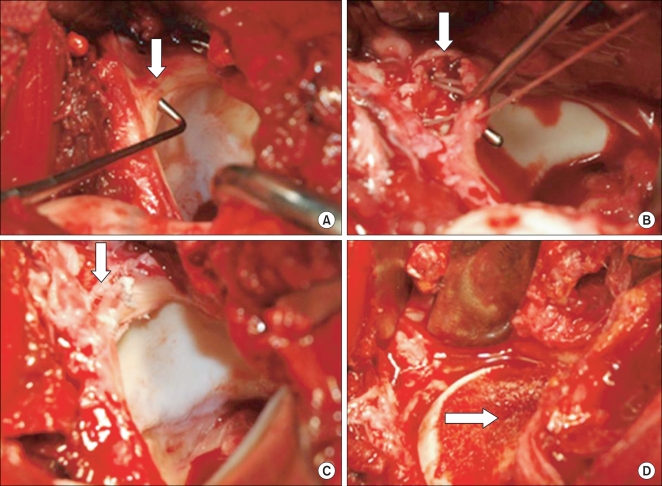
Intraoperative findings. (A) Labral tear was detected by a probe. (B) Acetabuloplasty of the anterosuperior of acetabular rim was performed. (C) Labral repair was done using an anchor suture. (D) Osteochondroplasty was performed at the  anterolateral side of the femoral head-neck junction.

**Fig. 4:**
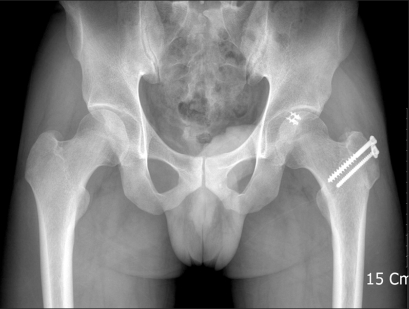
Postoperative radiograph shows union of the osteotomy site and no arthritic changes.

All procedures17) were carried out by the same surgeon (W.Y.S.). A 15-20 cm of straight-line skin incision was made with the patient in the lateral position. Along the anterior gluteus maximus and long axis of the femur, a fascia lata incision was performed to expose the greater trochanter. An osteotomy was performed taking care to maintain the gluteus medius, gluteus minimus, and vastus lateralis attached to the greater trochanteric fragment. A Z-shaped joint capsule incision was made with the fragment in anterior displacement and the hip in flexion, abduction, and external rotation. After eliciting an anterior dislocation of the femoral head and exposing the acetabular rim with a retractor, the cartilaginous lesion as well as the location and grade of the labral tear were inspected using a probe. The cartilage was irrigated intermittently with saline for protection and a dill hole was made in the femoral head-neck junction with a K-wire to check the blood supply. Acetabuloplasty was undertaken with an osteotome around the labral tear. The tear was repaired with suture anchors (2.8 mm, Fastak, Arthrex Inc., Naples, FL, USA) and osteochondroplasty was performed using a curved osteotome at the anterolateral side of the femoral-neck junction (Fig. 3). The dislocated hip was reduced. The presence of impingement was checked with the naked eye with the hip in flexion, adduction, and internal rotation. Closure of the articular capsule was performed taking care not to apply excessive tension. The greater trochanteric fragment was fixed with 2-3 bone screws. From the 2-3 postoperative day, the patients were allowed ≤ 70° of passive flexion exercise in order to prevent articular adhesion. For 6-8 weeks after surgery, progressive weight bearing ambulation was allowed depending on the union of the osteotomy site (Fig. 4). During the same period, the patients were asked to restrain from ≥ 70° of flexion and active abduction and adduction in order to promote bony union of the osteotomy site at the greater trochanter.

The radiological assessment was based on the pre- and postoperative measurements of the following parameters on the anteroposterior and lateral views of the hip: the degree of arthritis, Tönnis angle, center-edge angle of Wiberg, the presence of acetabular retroversion (cross-over sign and the posterior wall sign), femur neck-shaft angle, congruity of the femoral head and acetabulum, anterior femoral head-neck off set, and the presence of an abnormality at the femoral head (bump and herniation pit). The intra-articular abnormalities and alpha angle were assessed on the MR arthrograms. Cam type impingement was defined as the presence of a bump, herniation pit, and abnormal alpha angle. Pincer type impingement was defined as that of acetabular retroversion or protrusion. The mixed type impingement contained the features of the cam and pincer types. For a clinical assessment, the changes in the values of the impingement test and HHS measured preoperatively and at the last follow-up were analyzed.

The radiographic results showed a mean Tönnis angle (normal, ≤ 15°), which indicates the overcoverage of the anterosuperior acetalubum, of 7.9° (range, 5 to 16°), and was abnormally high in 2 cases. The mean center-edge angle of Wiberg (normal, ≥ 20°), which is also a measure of overcoverage of the anterosuperior acetalubum, was 35.5° (range, 17 to 48°), and decreased to < 20° in 1 case. In 6 cases, the cross-over and posterior wall signs27) were observed. The mean femur neck-shaft angle (normal range, 120 to 140°) was 131.3° (range, 118 to 141°), and was < 120° in 1 case and > 140° in another case. Asphericity of the femoral head was observed in 4 cases. The anterior femoral head-neck offset (normal, ≥ 7.2 mm), which is a measure of the distance between the femoral neck and acetabulum, is associated with FAI when it is abnormally small. The value was 12.6 mm (range, 6.4 to 17.2 mm) on average and small in 2 cases. With regard to abnormalities of the femoral head, a bump (a prominence at the femoral head-neck junction indicating impingement) was observed in 8 cases and a herniation pit was noted in 2 cases. Overall, 1 or more radiological abnormalities were identified in 12 cases (80%) (Table 1). Preoperatively, the Tönnis grade was 0, 1 and 2 in 7, 7 and 1 case, respectively. A comparison between the preoperative Tönnis grades and those obtained at the last follow-up showed that arthritis did not progress in any case. Only 14 MR arthrograms were evaluated because one was not available from 1 patient with a bilateral FAI. There were 11 cases (79%) of a labral tear, 6 cases (43%) of degenerative changes in the acetabular cartilage, and 3 cases (21%) of labral degeneration. The tear was located in the anterosuperior section in 9 cases (12 to 2 or 3 o'clock direction), in the anterosuperior and anteromedial section in 1 case (12 to 6 o'clock direction), and in the anterosuperior and posterosuperior section in 1 case (10 to 2 o'clock direction). Both labral tears and acetabular cartilage degeneration were observed in 3 cases and both labral degeneration and acetabular cartilage degeneration were noted in another 3 cases (Table 2). The alpha angle was measured in 11 patients excluding the first 3 patients in whom oblique axial MR arthrograms (coronal scout images) had not been obtained. The mean value was 61° (range, 32 to 105°). The value was > 50° in 8 cases (73%) and ≤ 50° in 3 cases (27%). With regard to the FAI type, there were 9 cam types and 6 mixed types.

## RESULTS

**Fig. 5:**
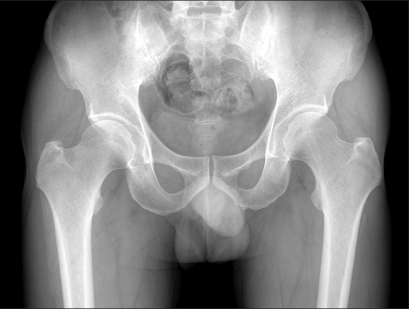
Both hip anteroposterior radiograph of a 46-year old male who suffered from left hip joint pain from 10 years ago shows a positive crossover sign in the right hip area.

**Fig. 6:**
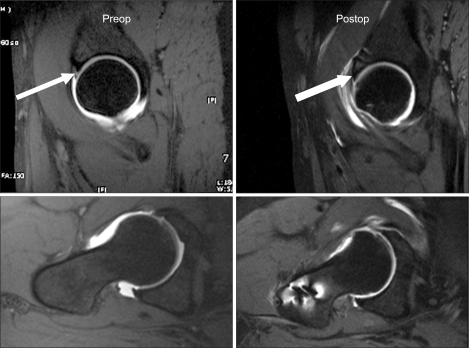
The pre- and postoperative MRIs shows a well-healed labral lesion and the amount of osteochondroplasty. In addition, there was no low-intensity signal, collapsed femoral head or double-line sign.

Intraoperatively, a labral tear was observed in 12 cases (80%). Eleven (79%) of them were noted in the MR arthrograms. Acetabular cartilage degeneration (soft ening, thinning, and fragmentation) was noted in 7 cases (43%). Labral ossification and acetabular osteophyte formation were identified in 3 (20%). Regarding the surgical techniques, a bumpectomy, labral repair, resection of the anterior rim and debridement was performed in 15, 12, 12 and 3 cases, respectively. The mean HHS improved from 76 points (range, 72 to 81 points) preoperatively to 93 points (range, 87 to 98 points) at the last follow-up. The anterior impingement test was negative in all cases at the last follow-up (Figs. 5 and 6).

No complications, such as avascular necrosis of the femoral head and infection developed at the last follow-up. However, there were 3 cases of nonunion of the trochanteric osteotomy site, which were treated with internal fixation using a trochanteric plate (Cable-Ready GTR, Zimmer, Warsaw, USA).

## DISCUSSION

Wenger et al.10) reported that structural abnormalities detectable by radiography were closely associated with labral tears in FAI. Accordingly, they concluded that identification of these structural abnormalities on radiographs could lead to an early diagnosis and optimal treatment planning of FAI. In the present study, 1 or more structural abnormalities were observed in 12 cases (80%) and 1 or more radiological abnormalities were noted in 11 cases, in which labral tears were detected on the MR arthrograms. Correlating the structural abnormalities with FAI can be controversial given the difficulty in obtaining precise and standardized radiographic images for the measurements as well as the lack of control group studies, and accuracy and objectivity studies. However, it is believed that the correlation can assist in terms of economy because the duration of symptoms was long in FAI patients and a relatively long time was needed to make a diagnosis. Indeed, the mean symptom duration in our study population was 2.3 years (range, 1 to 10 years).

MR arthrogram is the most reliable technique for detecting an intra-articular lesion, and allows an assessment of labral tears, labral cysts, cartilage degeneration, synovitis, and bony changes.^14^,19) The alpha angle measured on the oblique axial image is an accurate and reproducible parameter that is associated with cam type FAI if it is > 50°.28) In this study, all 11 cases of labral tears observed intraoperatively had also been diagnosed preoperatively by MR arthrography. Therefore, MR arthrography is an accurate diagnostic tool for labral tears. In this study, there were 9 anterosuperior tears, 1 anterosuperior and anteromedial tear, and 1 anterosuperior and posterosuperior tear. FAI was evident in the enrolled cases because the anterosuperior region was involved in all cases of labral tears. Although 9 cases were classified as the cam type FAI and 6 as the mixed type FAI in this study, radiography is a controversial technique in terms of objectivity and for detecting minor structural abnormalities. In contrast, the alpha angle is mostly associated with cam type FAI and can be measured from MRI. In addition, acetabular retroversion associated with pincer type FAI is also detectable using MRI,29) even though the acetabular retroversion could not be assessed by MR arthrogram on the affected side only. Therefore, it is believed that MRI (MR arthrogram), albeit expensive, is needed to classify the FAI type.

Labral tears in FAI are usually located in the anterosuperior region (12 to 2 or 3 o'clock direction). They tend to occur at the inner aspect of the labrum similar to partial rotator cuff tears, and rarely affect the entire layer. Like the meniscus, the labrum has a nerve endings for pain perception and proprioception, and pain occurs when it is damaged or under pressure.30) Biomechanically, labral damage results in an inadequate sealing effect, which also leads to early degeneration of the cartilage surface.22) In addition, natural recovery of the cartilage after treatment is rarely achieved when the accompanied cartilage degeneration is severe, causing rather persistent pain or degenerative arthritis.8) In this study, the labral tears found intraoperatively were intra-articular ones, and the cartilage degeneration assessed with the naked eye and a probe was classified as Outerbridge grade II or less in all cases. Therefore, no treatments for the degenerative cartilageous lesions (drilling, microfracture, and thermotherapy) were performed and the degeneration was restricted to the immediate vicinity of the labral tear or in the anterosuperior region. Arthritis did not progress postoperatively, and clinical improvement was observed at the last follow-up because the treatments were based on these intraoperative findings. Unfortunately, debridement instead of labral repair was needed in 3 cases with severe labral degeneration, which should be followed up to determine the risk of secondary arthritis. Furthermore, for an assessment of the prognosis, further studies will be needed on the grade and size of cartilage degeneration that can recover naturally.

Surgical dislocation, which is carried out with the hip in anterior dislocation, allows a large view field and easy detection of a lesion. It can be also considered as an alternative procedure in patients with FAI that is non-responsive to conservative treatment, particularly because procedures for common lesions in the anterosuperior acetabulum (acetabuloplasty, labral repair, drilling, microfracture, and thermotherapy) can be performed and assessed by the naked eye. However, assist devices, such as a spherometer gauge,3) may be necessary for a more accurate osteochondroplasty. Freccero et al.23) described a surgical dislocation as a safe procedure because no avascular necrosis of the femoral head was observed in postoperative MRI of 10 patients, who had underwent a surgical dislocation for FAI. In this study, although bone scans and MRI scans could not be performed in every case, blood flow in the femoral head was identified in all cases and avascular necrosis did not develop for after a clinical and radiological follow-up of more than 1 year. It is believed that a long-term follow-up study should be carried out for a precise assessment of avascular necrosis of the femoral head after a surgical dislocation for FAI. Indeed, reports have shown that avascular necrosis of the femoral head can occur in 2 post-traumatic years and even in 3 years in the case of a traumatic hip dislocation.

Nonunion at the trochanteric osteotomy site occurred as a complication in 3 cases (20%). This has not been reported previous studies^3^,^17^,^18^,23) on surgical dislocations17) performed in a similar manner. Therefore, it is suspected that an improper fixation technique, insufficient protection period might have been responsible for the postoperative complications. However, considering that most of the patients enrolled were young and active males, and only 2-3 bone screws were used for fixation, it is believed that the use of other fixation materials (claw plate, trochanteric plate, and supplementary wire ) should also be considered,13) even though there are concerns that these fixation materials may cause other complications (bursitis and metallosis).

To summarize, in a radiological assessment, 1 or more structural abnormalities were found in 12 cases (80%). Intraoperatively, 11 (79%) labral tears were detected on MR arthrograms. Therefore, radiographs and MR arthrogram are effective tools for diagnosis and treatment planning of patients with FAI. However, despite the satisfactory clinical outcomes of surgical dislocation, improvements in fixation methods at the trochanteric osteotomy site will be needed.

## Figures and Tables

**Table 1:**
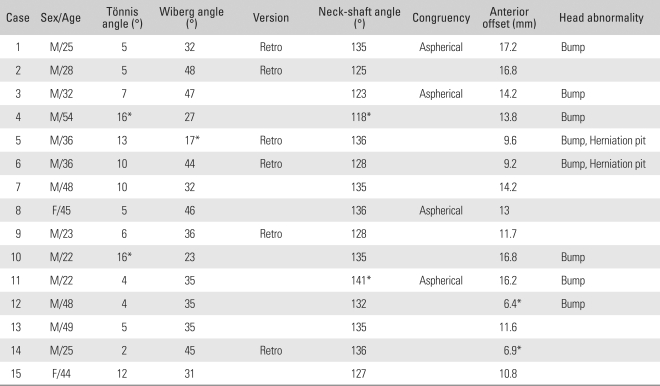
Results of the Radiographic Assessments

^*^Abnormal.

**Table 2:**
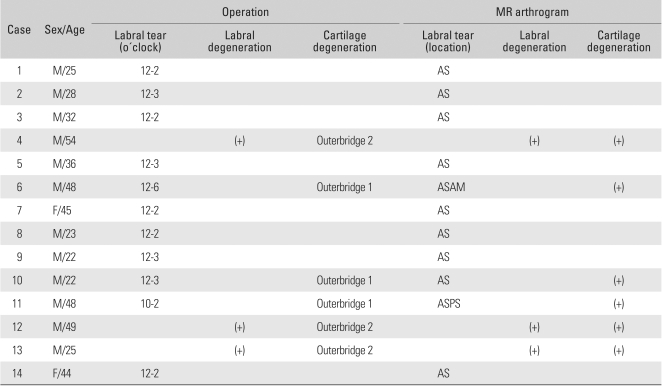
Comparison of the Findings between Surgery and MR Arthrogram

AS: Anterosuperior, ASAM: Anterosuperior and anteromiddle, ASPS: Anterosuperior and posterosuperior.
